# Deleting Mitochondrial Superoxide Dismutase 2 in Salivary Gland Ductal Epithelial Cells Recapitulates Non-Sjögren’s Sicca Syndrome

**DOI:** 10.3390/ijms25115983

**Published:** 2024-05-30

**Authors:** Joanna A. Papinska, Justyna Durślewicz, Harini Bagavant, Umesh S. Deshmukh

**Affiliations:** 1 Department of Microbiology and Immunology, Oklahoma University Health Sciences Center, Oklahoma City, OK 73104, USA; joanna-papinska@ouhsc.edu; 2 Arthritis and Clinical Immunology Program, Oklahoma Medical Research Foundation, Oklahoma City, OK 73104, USA; justyna.durslewicz@op.pl (J.D.); harini-bagavant@omrf.org (H.B.)

**Keywords:** mice, oxidative stress, salivary glands, sicca, Sjögren’s disease, superoxide dismutase 2

## Abstract

Elevated oxidative stress can play a pivotal role in autoimmune diseases by exacerbating inflammatory responses and tissue damage. In Sjögren’s disease (SjD), the contribution of oxidative stress in the disease pathogenesis remains unclear. To address this question, we created mice with a tamoxifen-inducible conditional knockout (KO) of a critical antioxidant enzyme, superoxide dismutase 2 (*Sod2)*, in the salivary glands (i-sg-*Sod2* KO mice). Following tamoxifen treatment, *Sod2* deletion occurred primarily in the ductal epithelium, and the salivary glands showed a significant downregulation of *Sod2* expression. At twelve weeks post-treatment, salivary glands from the i-sg-*Sod2* KO mice exhibited increased 3-Nitrotyrosine staining. Bulk RNA-seq revealed alterations in gene expression pathways related to ribosome biogenesis, mitochondrial function, and oxidative phosphorylation. Significant changes were noted in genes characteristic of salivary gland ionocytes. The i-sg-*Sod2* KO mice developed reversible glandular hypofunction. However, this functional loss was not accompanied by glandular lymphocytic foci or circulating anti-nuclear antibodies. These data demonstrate that although localized oxidative stress in salivary gland ductal cells was insufficient for SjD development, it induced glandular dysfunction. The i-sg-*Sod2* KO mouse resembles patients classified as non-Sjögren’s sicca and will be a valuable model for deciphering oxidative-stress-mediated glandular dysfunction and recovery mechanisms.

## 1. Introduction

Sjögren’s disease (SjD) is a chronic autoimmune disorder that disproportionally affects women (9:1 ratio compared to men) and is mainly diagnosed in the postmenopausal age group. Therefore, sex and age are considered significant risk factors in disease development [1]. Oxidative stress, associated with aging and hormonal changes, results from the imbalance between reactive oxygen species (ROS) generation and the antioxidant machinery. ROS generated during normal respiration are characterized by their high reactivity with different biomolecules [2]. As an essential component of cell signaling, ROS eradicate infections and activate numerous physiological pathways [3]. Excessive ROS accumulation leads to the dangerous modification of cell components, including proteins, lipids, and nucleic acids, causing cellular damage. Products of ROS modifications can also serve as danger-associated molecular patterns and initiate sterile inflammation [3,4]. Thus, ROS may lead to inflammation and further tissue injury by their direct or indirect actions.

Salivary glands are exposed to many factors, like viruses and bacteria, substances such as alcohol and tobacco byproducts, and potentially harmful ingredients in medications. This exposure poses a persistent challenge for maintaining homeostasis, ultimately increasing the risk of oxidative stress [5]. SjD patients have elevated levels of oxidative stress biomarkers in circulation, conjunctival epithelial cells, minor labial salivary gland biopsy, saliva, serum, and plasma [6,7,8,9,10,11,12]. These biomarkers are indicative of DNA damage (8-OHdG), lipid peroxidation (HEL and 4-HNE), and protein nitration (nitrotyrosine). In addition, reduced antioxidant enzyme levels leading to disturbed homeostasis may contribute to elevated oxidative stress in SjD patients [13]. These findings, considered together, suggest the possible involvement of oxidative stress in SjD pathogenesis.

In SjD patients, salivary gland hypofunction leads to reduced fluid secretion and often manifests as dry mouth [14]. Immune cell infiltration and formation of lymphocytic foci in salivary glands is a characteristic feature of SjD. However, there is a lack of correlation between the severity of glandular inflammation and the magnitude of glandular hypofunction [15]. Moreover, in a group of patients with dry eye and dry mouth defined as non-Sjögren’s sicca syndrome, glandular hypofunction occurs without the presence of organized lymphocytic foci in the salivary glands [16,17]. These observations suggest that multiple immune and non-immune mechanisms must contribute to glandular hypofunction in SjD patients.

The functional unit of the salivary gland includes the acinar and ductal cell compartments. Acinar cells are responsive to neural signals, and saliva production involves the flow of water and electrolytes via the transcellular and paracellular transport mechanisms and the synthesis and secretion of proteins. The ductal cells, on the other hand, play a crucial role in modifying the ionic composition of saliva [18]. The salivary gland function is under the precise and constant control of the autonomic nervous system (ANS). Under normal conditions, ANS dynamically balances the parasympathetic and sympathetic stimulation. Upon stress conditions, the sympathetic branch of the ANS is activated, reducing saliva secretion [19,20]. Certain medications known as xerogenic drugs can exacerbate these effects, leading to a decrease in salivation. These medications include various classes of drugs: antihistamines, antidepressants, and antihypertensives, which interfere with the normal cholinergic pathways that facilitate salivation [21,22]. Overall, stress or medications can potentially disrupt secretory processes maintained by the ANS. In SjD, the influence of oxidative stress on glandular function is unknown.

In this study, we hypothesized that elevated oxidative stress in salivary glands would lead to cellular damage and activation of innate immunity, causing glandular inflammation similar to that noted in patients with SjD. The combined effects of localized oxidative stress and an ensuing autoimmune response would lead to the development of SjD. To address this hypothesis, we developed mouse models that genetically target one of the critical mitochondrial pathways involved in eliminating free oxygen radicals. Contrary to our expectations, despite the induction of localized oxidative stress, the mice did not develop SjD. Instead, the phenotype in these mice resembled non-Sjögren’s sicca patients.

## 2. Results

### 2.1. Sod2 Expression Is Reduced in Salivary Glands of i-sg-Sod2 KO Mice

To induce localized oxidative stress in salivary glands, we created *Sod2^fl/fl^*; *Tfcp2l1^cre/ERT2^* mice (i-sg-*Sod2* KO) by crossing *Sod2^fl/fl^* [23] mice with *Tfcp2l1^cre/ERT2^* mice [24]. *Cre*-negative littermates were used as controls in all experiments. Following tamoxifen treatment, salivary glands of i-sg-*Sod2* KO mice showed a significant reduction in *Sod2* expression (Figure S1). In salivary glands, the transcription factor *Tfcp2l1* is only expressed in the ductal cells (Figure S2) [24], which may explain the limited loss of *Sod2* gene expression.

**Figure 1:**
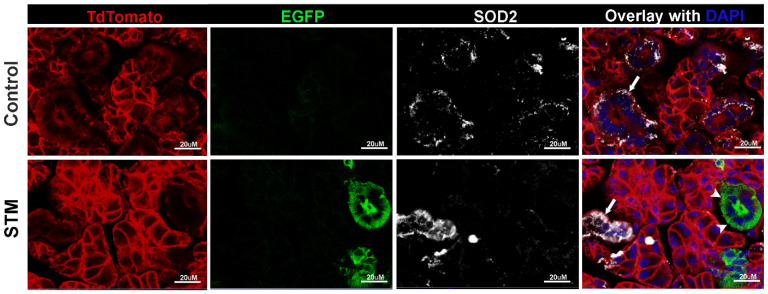
Lack of SOD2 staining in ductal cells of STM mice. Representative immunofluorescence images of SOD2 staining in the submandibular salivary glands from tamoxifen-treated control mice (*Sod2^fl/fl^*; *Rosa26R-mT/mG*) (**upper panel**) and STM (*Sod2^fl/fl^*; *Tfcp2l1^cre/ERT^*; *Rosa26R-mT/mG)* mice (**bottom panel**). Green EGFP fluorescence (arrowheads) within the cells indicates Crerecombinase activity. These cells do not show SOD2 (white) expression. Cells without Cre–recombinase activity show TdTomato (red) expression and stain for SOD2 (arrows). Nuclear staining with DAPI is shown in blue. Scale bars = 20 μm.

To confirm ductal-specific ablation of *Sod2*, we generated *Sod2^fl/fl^*; *Tfcp2l1^cre/ERT2^*; *Rosa26R-mT/mG* (STM) mice. Salivary glands of tamoxifen-treated control mice, *cre/ERT2* negative littermates (*Sod2^fl/fl^*; *Rosa26R-mT/mG)*, did not show any EGFP^+^ cells, and SOD2 was prominently detected in the TdTomato^+^EGFP^−^ ductal cells of these mice (Figure 1, upper panel). In contrast, after tamoxifen treatment, STM salivary glands showed EGFP expression in the ductal cells, indicating Cre-mediated recombination in these cells (Figure 1, bottom panel). The EGFP^+^ ductal cells in STM mice did not stain for SOD2 protein. These data establish that conditional *Sod2* deletion in salivary glands in our mouse models occurs in ductal epithelial cells.

### 2.2. Deletion of Sod2 in the Ductal Epithelial Cells Leads to an Increase in Oxidative Stress

**Figure 2:**
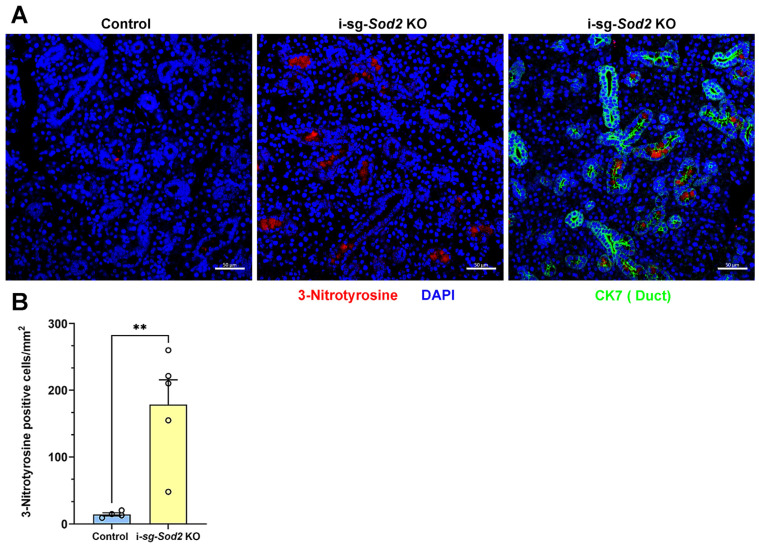
Elevated oxidative stress in salivary gland ductal cells from i-sg-*Sod2* KO mice. (**A**) Representative images of nitrotyrosine staining (red) in submandibular salivary glands from i-sg-*Sod2* KO (middle and right panels) and littermate control mice (left panel), 12–13 weeks post-tamoxifen treatment. The right panel shows cytokeratin 7 (CK7)-positive ductal cells (green). Nuclei (blue) stained with DAPI. Scale bars = 50 μm. (**B**) Quantification of nitrotyrosine-positive cells per square mm of the tissue section. Data are presented as mean + standard error of the mean (SEM). Statistical significance was determined using Student’s *t*-test (** *p* < 0.01).

To investigate whether the downregulation of *Sod2* expression in the salivary glands leads to increased oxidative stress, salivary glands from i-sg-*Sod2* KO mice were stained with an anti-nitrotyrosine antibody. A significantly elevated number of nitrotyrosine-positive cells were detected in the salivary glands of i-sg-*Sod2* KO mice compared to the control mice (Figure 2). The nitrotyrosine positivity was restricted to cytokeratin 7 (CK7)-expressing ductal cells. These data suggest that *Sod2* deletion in salivary gland ductal cells leads to elevated oxidative stress in these cells.

### 2.3. RNA-Seq Analysis of i-sg-Sod2 KO Salivary Glands Shows Significant Alterations in Gene Expression

**Figure 3:**
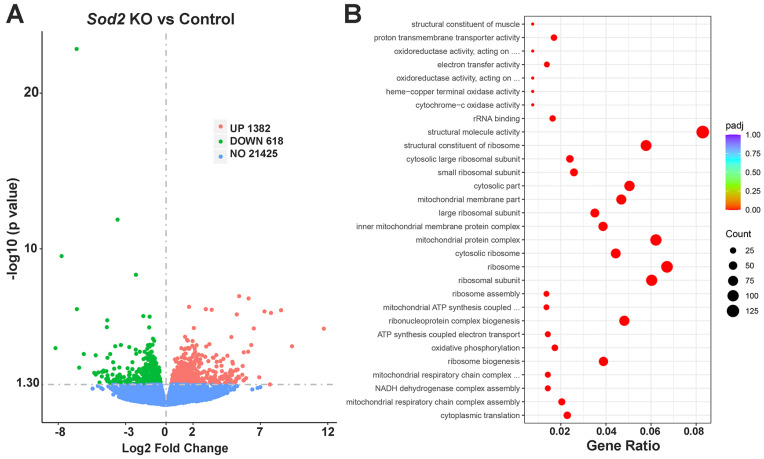
Analysis of bulk RNA-seq data from salivary glands of i-sg-*Sod2* KO. (**A**) The volcano plot illustrates the differential gene expression profile of i-sg-*Sod2* KO mice vs. their control littermates, *n* = 3 per group. Each point represents a gene, either upregulated (pink) or downregulated (green) in KO mice. The horizontal dashed line represents the threshold for statistical significance (adjusted *p*-value < 0.05). (**B**) Gene ontology (GO) pathway enrichment analysis representing prominently affected biological pathways in salivary glands from i-sg-*Sod2* KO mice. The dot size corresponds to the number of genes associated with the pathway, and the color indicates the adjusted *p*-value. The complete heading of pathways with …. designation on y-axis from top to bottom are: oxidoreductase activity acting on a heme group of donors; oxidoreductase activity acting on peroxide as acceptor; mitochondrial ATP synthesis coupled electron transport; mitochondrial respiratory chain complex I assembly.

To investigate which pathways were affected by *Sod2* deletion, salivary gland RNA from i-sg-*Sod2* KO and littermate controls were isolated and subjected to bulk RNA-seq. Significant changes in the gene expression profile were noted between the i-sg-*Sod2* KO and control mice. Overall, 2000 genes were differentially expressed, with 1382 genes upregulated and 618 genes downregulated in the i-sg-*Sod2* KO mice (Figure 3A). The three most significantly downregulated genes in the salivary glands of i-sg-*Sod2*-KO mice were *Smgc* (log_2_Fold change: −3.59, *p* = 1.30 × 10^−12^), *Hapln4* (log_2_Fold change: −7.74, *p* = 2.93 × 10^−10^), and *Pon1* (log_2_Fold change: −2.23, *p* = 4.58 × 10^−9^). Of these, only *Smgc* has prominent expression in the salivary glands, whereas *Hapln4* and *Pon1* are enriched for expression in the CNS and liver, respectively [25,26]. The *Smgc* gene encodes for the submandibular gland protein C and is located on chromosome 15 within the *Smgc/Muc19* gene complex [27]. Alternate splicing leads to the production of either SMGC or MUC19 proteins [28]. The SMGC/MUC19 are large gel-forming secreted mucins that maintain oral health through lubrication and microbial clearance [29]. In adult female mice, *Smgc* expression in salivary glands is mainly restricted to terminal tubule cells [30]. Thus, low expression of *Smgc* in salivary glands from i-sg-*Sod2*-KO mice indicates altered gene expression in ductal cells, possibly undergoing oxidative stress. The top three significantly upregulated genes in the salivary glands of i-sg-*Sod2*-KO mice were *Mylpf* (Log_2_Fold change: 5.42, *p* = 1.08 × 10^−7^), *Actn3* (Log_2_Fold change: 6.11, *p* = 1.50 × 10^−7^), and *Tnnt3* (Log_2_Fold change: 8.54, *p* = 8.39 × 10^−7^). *Mylpf*, *Actn3*, and *Tnnt3* encode for myosin regulatory light chain 11, alpha-actinin-3, and troponin T fast skeletal muscle proteins, respectively. All three proteins are involved in cytoskeleton organization and are structural constituents of muscle. However, the biological significance of elevated expression of these three genes in salivary glands undergoing oxidative stress is unclear.

**Figure 4:**
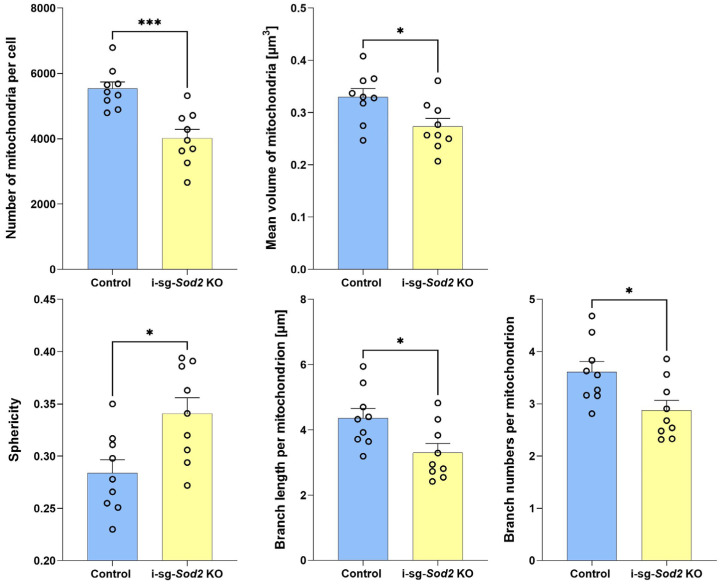
Analysis of mitochondrial morphology in salivary gland ductal cells of i-sg-*Sod2* KO mice. Salivary gland tissue sections from i-sg-*Sod2* KO (*n* = 3) and littermate control mice (*n* = 3) were stained with mitochondrial marker, MFF at 12–13 weeks post-tamoxifen treatment. Z-stack images were captured from three distinct areas per section; each data point represents a single Z-stack image analysis. Data are presented as mean + SEM. Statistical significance was determined using Student’s *t*-test (*** *p* < 0.001, * *p* < 0.05).

Enrichment analysis using gene ontology (GO) demonstrated that several pathways involving ribosome biogenesis, assembly, and structure were significantly affected. Considering that SOD2 is a mitochondrial protein, as expected, pathways related to mitochondrial structure and function were influenced by *Sod2* deletion (Figure 3B). To address the possible effects of *Sod2* deletion on mitochondrial structure, formalin-fixed, paraffin-embedded salivary gland tissue sections were stained with anti-mitochondrial fission factor (MFF) antibody (Figure S3). Significant changes were noted in mitochondrial morphology between ductal cells from i-sg-*Sod2*-KO mice and control littermate mice (Figure 4). The number of mitochondria was significantly lower in the i-sg-*Sod2*-KO mice. Moreover, the mitochondria from these mice had increased sphericity and, conversely, decreased number and length of branching. These data suggest that *Sod2* deletion in salivary gland ductal cells significantly influences mitochondrial structure in these cells.

**Figure 5:**
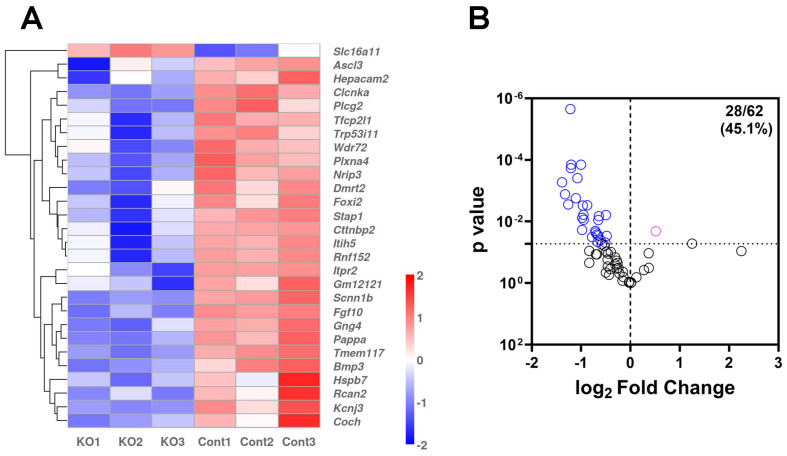
Reduced expression of genes associated with salivary gland ionocytes. (**A**) Heatmap of the ionocyte-related differentially expressed genes between i-sg-*Sod2* KO mice (KO) and littermate controls (Cont). (**B**) A volcano plot of ionocyte genes in the i-sg-*Sod2* KO mice versus control mice. Among the 62 genes associated with ionocytes, 28 were differentially expressed (blue: downregulation, pink: upregulation, black: not significant at *p* < 0.05).

The salivary gland gene expression atlas [31] shows that the *Tfcp2l1* gene, used for *cre/ERT2* expression in the i-sg-*Sod2* KO mice, is highly expressed in ASCL3+ ductal cells. Salivary gland ASCL3+ cells have been previously described as progenitors for acinar and ductal cells [32]. However, a recent report shows that ASCL3+ ductal cells also represent salivary gland ionocytes and contribute to ion transport and salivary gland homeostasis [33]. Some genes uniquely expressed in ionocytes, including *Ascl3*, *Foxi2*, and *Stap1*, were significantly downregulated in i-sg-*Sod2* KO mice (Figure 5A). Overall, out of the 62 genes that are known to be associated with salivary gland ionocytes [33], 28 genes were differentially expressed in the i-sg-*Sod2* KO mice (*p* < 0.05). Of the 28 differentially expressed ionocyte genes, all except only 1 (*Slc16a11*) were downregulated (Figure 5B).

### 2.4. I-sg-Sod2 KO Mice Develop Salivary Gland Dysfunction without Significant Differences in Tissue Pathology

**Figure 6:**
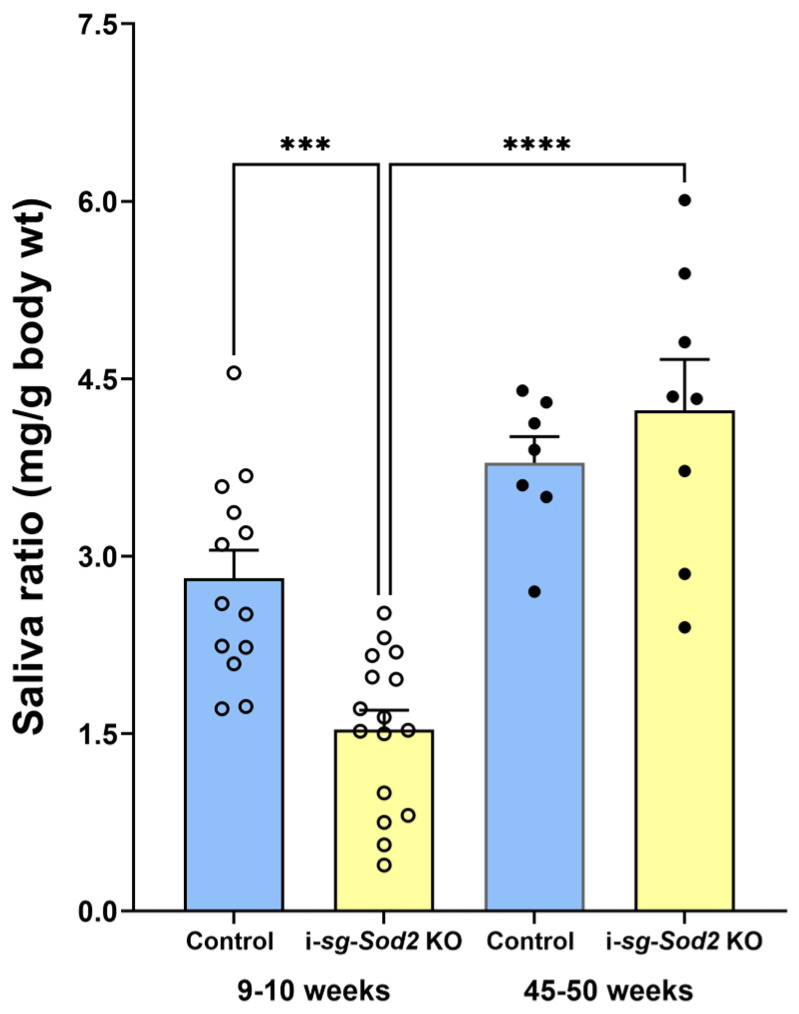
Glandular hypofunction in i-sg-*Sod2* KO is reversible. Pilocarpine-induced saliva was measured in i-sg-*Sod2* KO mice and their littermate controls, 9–10 weeks and 45–50 weeks post-tamoxifen treatment. At 9–10 weeks, the mean amount of saliva in i-sg-*Sod2* KO mice was significantly lower than in their littermate controls. However, at 45–50 weeks, the mean saliva amount between the two groups was comparable, and the recovery of glandular hypofunction in i-sg-*Sod2* KO was significant. Statistical significance was determined by one-way ANOVA and Sidak’s post-test for multiple comparisons. *p* < 0.05 was considered significant. *** *p* < 0.001, **** *p* < 0.0001.

Reduced saliva production and dry mouth are characteristic features of SjD. Pilocarpine-induced saliva was measured to evaluate glandular function in i-sg-*Sod2* KO mice. At nine weeks after tamoxifen treatment, i-sg-*Sod2* KO mice produced significantly less saliva than the littermate control mice (Figure 6). Saliva production was studied 45–50 weeks post-tamoxifen treatment to determine the long-term effect of ductal *Sod2* deficiency. Surprisingly, salivary function was wholly recovered in the i-sg-*Sod2* KO mice, and saliva production was comparable to age-matched littermate controls. Compared to the early time point, littermate controls also showed a slight increase in saliva production at 45–50 weeks post-tamoxifen. However, the differences were not significant.

**Figure 7:**
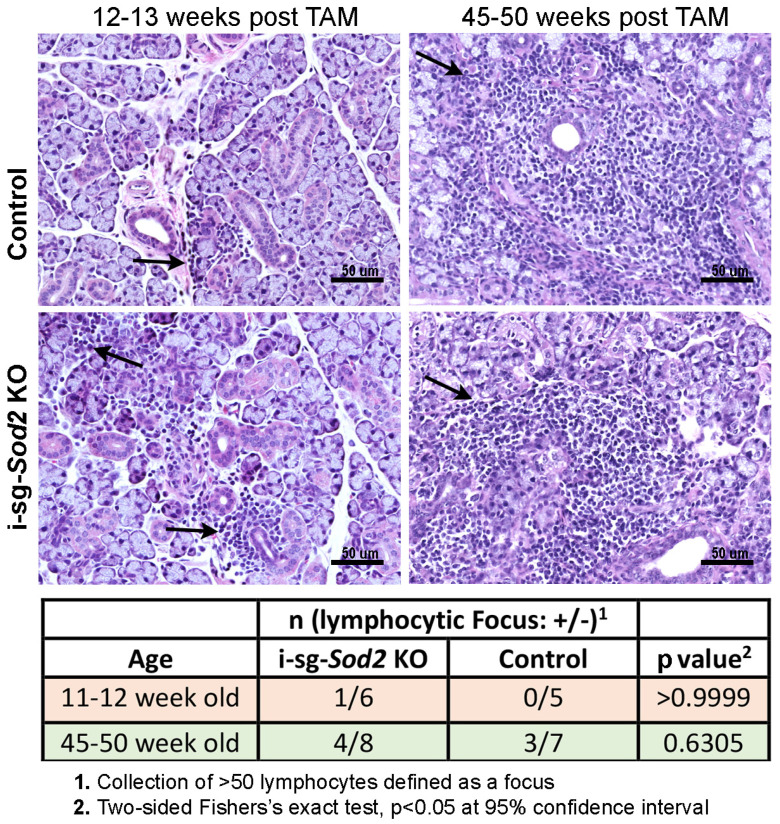
Analysis of lymphocytic foci in submandibular salivary glands of i-sg-*Sod2* KO and control mice. **Top panel**: images of H-and-E-stained sections of formalin-fixed, paraffin-embedded submandibular salivary gland tissues from i-sg-*Sod2* KO and control mice at 12–13 (left) and 45–50 weeks (right) post-treatment. Mice with the most severe inflammation at each time point are shown. Arrows point to foci of inflammation. **Bottom panel**: No significant differences were present between both groups of mice, regardless of the time point.

Lymphocytic foci within the minor salivary glands are a significant classification criterion for SjD. To evaluate whether i-sg-*Sod2* KO mice develop lymphocytic foci, salivary gland tissues were harvested from mice, and tissue sections were stained with hematoxylin and eosin (Figure 7). In both groups, at 12–13 weeks, most mice did not develop classic foci of inflammation. In i-sg-*Sod2* KO mice, only one mouse (out of seven) showed severe peri-ductal inflammation. Although at the later time point (>45 weeks post), the frequency and severity of inflammation were higher in the i-sg-*Sod2* KO mice, this was not significantly different from the control littermates.

## 3. Discussion

In this study, to investigate the possible role of elevated salivary gland oxidative stress in SjD development, we conditionally deleted the *Sod2* gene in salivary gland ductal epithelial cells. The mitochondrial SOD2 enzyme plays a crucial role in the cell’s antioxidant defense mechanism by converting superoxide radical anion to hydrogen peroxide, which is further converted by the enzyme catalase to water and molecular oxygen [34]. Lack of SOD2 results in the accumulation of highly reactive superoxide radicals and severe cellular damage [35]. The germline deletion of the *Sod2* gene yields very severe phenotypes (dilated cardiomyopathy, hypothermia, growth retardation, and accumulation of lipids in the liver) [36]. Consequently, mice develop multi-systemic dysfunction and, depending on their genetic background, typically die between 10–21 days [23,37]. Due to this detrimental effect of constitutive *Sod2* deletion, multiple conditional, tissue-specific knockouts of *Sod2* have been created to delineate its function *in vivo* [38,39,40]. Therefore, our study used a *Tfcp2l1* promoter-driven *cre/ERT2* line [24] to conditionally knock out *Sod2* and induce oxidative stress in salivary glands. In agreement with the previously reported expression of *Tfcp2l1* [24,31], the *cre/ERT2* activity in the salivary glands was prominently localized to ductal epithelial cells (Figure S2). Tamoxifen-induced deletion of *Sod2* in ductal cells increased nitrotyrosine positivity within these cells, providing evidence of elevated localized oxidative stress in the salivary glands.

Bulk RNA-seq of salivary glands at 12–13 weeks post-tamoxifen treatment showed altered expression of several genes. Based on the literature, we hypothesized that mitochondrial oxidative stress would cause mitochondrial damage and the activation of the cGAS-STING pathway, leading to type I IFN, pro-inflammatory cytokine production and SjD development [41]. Contrary to our expectations, we did not see significant changes in immune pathway genes, particularly those involved in the type I IFN signature. Instead, pathways involved in cellular metabolism, oxidative phosphorylation, and ribosomal function were affected. The changes in gene expression in mitochondrial pathways were reflected in the altered number and structure of mitochondria in ductal cells of i-sg-*Sod2* KO mice. Whether these changes reflect an oxidative-stress-induced imbalance in mitochondrial fission versus fusion and how this influences mitochondrial function will be studied in the future.

In the i-sg-*Sod2* KO mice, the downregulation of several genes linked with the salivary gland ionocyte population was of note. It has been recently demonstrated that following radiation-induced damage, salivary gland ionocytes might have a critical role in restoring the function of the salivary glands [33]. Salivary gland ionocytes secrete fibroblast growth factor (FGF-10), which acts as an essential molecule in the development and renewal of salivary gland cells, thereby influencing the ability of the tissue to regenerate. Although the primary function attributed to salivary gland ionocytes is maintaining the ionic composition of saliva, whether they contribute directly or indirectly to saliva production is unknown. A recent report has suggested that intercalated duct cells, which were assumed to only function in maintaining the ionic composition of saliva, have properties of secretory cells and might contribute towards saliva production [42]. The i-sg*-Sod2* KO model will be a valuable tool for further investigating the role of ionocytes in salivary gland function.

The i-sg*-Sod2* KO mice demonstrated evidence of glandular hypofunction. At 9–10 weeks post-tamoxifen treatment, these mice produced significantly lower amounts of saliva than the control littermates. However, over time, the glandular hypofunction recovered, and at 45–50 weeks post-tamoxifen treatment, the difference in mean saliva volumes between the i-sg*-Sod2* KO mice and control littermates was statistically not significant. The mechanisms involved in glandular hypofunction and recovery in i-sg*-Sod2* KO mice are unknown. It would be of interest to ask the question, how does oxidative stress primarily induced in ductal epithelial cells influence pilocarpine-induced fluid secretion? In this context, a previous study has reported that exposing mice for only 5 days to hyperoxic conditions (75% O_2_) induced oxidative stress and glandular hypofunction [43]. While functional recovery was not evaluated in the hyperoxic mice, our study shows that oxidative-stress-induced hypofunction in young mice can be restored. Possible mechanisms include the compensatory activation of antioxidant pathways, which need to be explored.

At an early time point, lymphocytic foci, characteristic of SjD, were not observed in the salivary glands of i-sg*-Sod2* KO mice. At the later time point, both i-sg*-Sod2* KO and control littermates showed higher severity and incidence of sialadenitis. Recently, we have reported that aged mice develop lymphocytic foci in their salivary glands, similar to those seen in SjD patients [44]. Thus, aging might contribute to lymphocytic foci in old i-sg-*Sod2* KO mice and control littermates.

Although dry mouth and dry eye are prominent features of SjD, to be classified as an SjD patient, a positive minor labial gland biopsy (focus score ≥ 1.0) and the presence of autoantibodies like anti-Ro are required [45]. At the time of glandular hypofunction, the i-sg-*Sod2* KO mice did not have a positive biopsy focus score or the presence of circulating anti-Ro or anti-nuclear antibodies. Thus, the i-sg*-Sod2* KO mice mimic non-Sjögren’s sicca patients. These patients suffer from dryness unrelated to medication use or other underlying autoimmune disorders [46]. The non-Sjögren’s sicca patients do not present with autoimmunity and do not fulfill the classification criteria for SjD, thereby leading to their exclusion from clinical trials designed for SjD. Considering that the non-Sjögren’s sicca patients report poorer oral health and lower health-related quality of life than patients with SjD [47], mechanisms driving dryness in these patients need to be investigated. Our findings suggest a plausible thesis that elevated oxidative stress in salivary glands might contribute to dryness in non-Sjögren’s sicca patients.

There is an increased interest in exploring antioxidant therapies for SjD treatment [48]. Indeed, in a clinical study, supplementation with Pycnogenol, a well-known antioxidant from a pine tree, improved symptoms of eye and mouth dryness [49]. Thus, it is certainly possible that non-Sjögren’s sicca patients might also benefit from therapies aimed at reducing oxidative stress.

## 4. Materials and Methods

### 4.1. Mouse Models

All animal work was approved by the Institutional Animal Care and Use Committee (IACUC) of the Oklahoma Medical Research Foundation (OMRF), and all procedures followed the guidelines and regulations established by the National Institutes of Health. All experiments were designed to minimize the number of animals used. Mice had unrestricted access to food and water and were fed a PicoLab standard 5053 diet (LabDiet, Richmond, IN, USA).

### 4.2. Generation of Sod2^fl/fl^; Tfcp2l1^cre/ERT2^ Mice

Female *Sod2^fl/fl^* mice [23] kindly provided by Dr. Holly van Remmen (OMRF) were crossed with *Tfcp2l1^cre/ERT2^* mice [24] purchased from the Jackson laboratory, Bar Harbor, ME, USA (JAX stock #0287320) to generate (*Sod2^fl/−^* × *Tfcp2l1^cre/ERT2^*^+/−^) F1 mice. The F1 mice were backcrossed with the *Sod2^fl/fl^* mice to generate *Sod2^fl/fl^*; *Tfcp2l1^cre/ERT2^* (i-sg-*Sod2* KO) mice. *Sod2^fl/fl^*; *Tfcp2l1^cre/ERT2^*^−/−^ littermate mice were used as controls.

### 4.3. Generation of Sod2^fl/fl^; Tfcp2l1^cre/ERT2^; Rosa26R-mT/mG^+/−^ Mice

The double heterozygous mice (*Sod2^fl/+^*; *Tfcp2l1*^cre/ERT2+/−^) were intercrossed with *Rosa26R-mT/mG* reporter mice [50] purchased from the Jackson Laboratory (JAX stock #007676) to generate *Sod2^fl/+^*; *Tfcp2l1*^cre/ERT2+/−^; *Rosa26R-mT/mG^+/−^* mice. These triple heterozygous mice were backcrossed with *Sod2^fl/fl^* mice to obtain mice homozygous for *Sod2^fl/fl^*, heterozygous for *Tfcp2l1*^cre/ERT2^, and heterozygous for *Rosa26R-mT/mG^+/−^* (*Sod2^fl/fl^*; *Tfcp2l1^cre/ERT2^*; *Rosa26R-mT/mG)* (STM reporter mice). In STM reporter mice, tamoxifen treatment induced Cre-mediated recombination, resulting in the deletion of *Sod2* and the expression of membrane-bound EGFP, causing the cells to emit green fluorescence. Cells lacking Cre–recombinase activity would express membrane-bound TdTomato, emitting red fluorescence.

### 4.4. Tamoxifen Treatment

Female mice at 8–10 weeks of age were treated with tamoxifen per protocols recommended by the Jackson laboratory. Briefly, tamoxifen (Glpbio, Montclair, CA, USA) dissolved in corn oil (20 mg/mL) was injected consecutively by an intraperitoneal route for 5 days at a 75 mg/kg body weight dose.

### 4.5. Saliva Collection

Saliva production induced by pilocarpine was measured in mice following the method previously described [51]. Following anesthesia, mice received an intraperitoneal injection of pilocarpine hydrochloride (0.375 mg/kg body weight). Saliva was collected by inserting an absorbent sponge (Salimetrics, Carlsbad, CA, USA) into the animal’s mouth for 15 min. The weight of saliva produced was determined by calculating the difference between the wet and dry weight of the sponge.

### 4.6. Immunohistochemistry and Immunofluorescence

*mT*/*mG* visualization. Mice were perfused with phosphate buffer saline (PBS), and submandibular glands were fixed in 1% paraformaldehyde–lysine–periodate (PLP) for 24 h at 4 °C, followed by storage in 30% sucrose with 0.1% sodium azide until further use. For cryosectioning, tissues were embedded in OCT, and 10 μm sections were obtained on CryoStar NX50 Cryostat (Epredia, Kalamazoo, MI, USA). Slides were dried overnight, followed by 3× PBS wash. Nuclei were stained using DAPI, and coverslips were mounted using Prolong Gold (Thermofisher, Waltham, MA, USA). Images were acquired using LSM710 microscope and Zen software, 3.0 Blue Edition (Carl Zeiss Microscopy LLC, White Plains, NY, USA).

SOD2 staining. Submandibular salivary glands were dissected and processed following the same procedure as for *mT*/*mG* visualization. Slides were subjected to a 15-min incubation with 0.3% TritonX-100 in PBS, followed by PBS washing (3 × 5 min) and a blocking step with normal horse serum (1:50 dilution) in 1% BSA with 0.1% TritonX-100 for 15 min at room temperature. Tissue sections were incubated with a 1:100 dilution of rabbit anti-SOD2 antibody (Proteintech, Rosemont, IL, USA) at room temperature for 4 h. Subsequently, after a washing step (3 × 5 min), slides were incubated with AF647-conjugated donkey anti-rabbit antibody (Jackson ImmunoResearch Labs, West Grove, PA, USA) for 1 h at RT in the dark. Nuclei were stained with DAPI, followed by another round of washing (3 × 5 min), and coverslips were mounted using Prolong Gold. Images were acquired using an LSM710 confocal microscope and Zen software, 3.0 Blue Edition. 

Haematoxylin and Eosin (H and E) staining. Tissue sections (5 μm thickness) from formalin-fixed and paraffin-embedded submandibular glands were stained with H and E, as described previously [44].

Nitrotyrosine and Cytokeratin 7 staining. Five-micron tissue sections from formalin-fixed and paraffin-embedded submandibular glands underwent acidic antigen retrieval with citric acid, as described previously [52], and were stained with rabbit anti-nitrotyrosine antibody (Thermofisher, Waltham, MA, USA) using the protocol described above for SOD2 staining, except that the incubation was overnight at 4 °C. Bound antibody was detected by using AF647-conjugated donkey anti-rabbit IgG antibody. Nuclei were stained with DAPI, and the slides were mounted in Prolong Gold. Images were captured using an LSM710 microscope and Zen software, 3.0 Blue Edition. QuPath software, Version 0.4.3 [53] was used for image analysis to quantify the nitrotyrosine-positive signal. Images were captured from 3 different regions within salivary glands, and cells positive for nitrotyrosine were recognized by the positive cell detection tool in the QuPath software, Version 0.4.3. Alexa Fluor 488-conjugated anti-CK7 antibody (Bioss Antibodies, Woburn, MA, USA) was used for cytokeratin 7 staining, and the above-described protocol was used.

Mitochondrial Fission Factor (MFF) staining. Three-micron tissue sections from formalin-fixed and paraffin-embedded submandibular glands were processed according to the protocol described above for Nitrotyrosine and Cytokeratin 7 staining. Z-stack images were captured on an LSM710 confocal microscope, 63× (NA 1.40) objective, Pin Hole 1.0 AU and Z Step, and 0.364 μm and Zen software, 3.0 Blue Edition. Mitochondrial morphology was further assessed using Fiji/ImageJ software (https://fiji.sc/) with the Mitochondria Analyzer plugin, which analyzes mitochondrial shape, size, and cell distribution. Briefly, images of tissue sections stained with anti-MFF (Cell Signaling Technology, Danvers, MA, USA) were uploaded to Fiji/ImageJ. For each image, a region of interest (ROI) composed of salivary gland ducts was defined, and the Mitochondria Analyzer plugin was then used to analyze mitochondria within a specified ROI. The parameters examined included mitochondria count per cell, the mean volume of mitochondria, sphericity, length of mitochondrial branches, and number of branches per mitochondrion.

### 4.7. Gene Expression Analysis

Following the dissection of salivary glands from mice, tissues were immediately frozen in liquid nitrogen and stored at −80 °C until further use. Total RNA from submandibular glands was extracted using the RNeasy Mini Kit (Qiagen, Germantown, MD, USA) according to the protocol provided by the manufacturer. *Sod2* expression level in submandibular glands was analyzed by real-time polymerase chain reaction using TaqMan assays (Thermofisher, Waltham, MA, USA) as previously described [54]. *Hprt1* was used as a housekeeping gene for data normalization.

RNA-seq (library preparation, quality control, and 150 bp paired-end sequencing on Illumina platform) and bioinformatics analysis describing differential gene expression and pathway analysis were performed by Novogene (Sacramento, CA, USA). Additional data analysis was performed using Novomagic, a free Novogene platform for data analysis (Novogene, Sacramento, CA, USA).

### 4.8. Statistical Analysis

Statistical analyses were performed using Prism 9.4 software (GraphPad Software, Boston, MA, USA). The normality test was used for each dataset to determine Gaussian distribution. Students’ *t*-test was used to determine the differences between groups in the data following a Gaussian distribution. For non-Gaussian distribution, a non-parametric Mann–Whitney test was used. One-way ANOVA with Sidak’s post-test was used for multiple group comparisons. A *p* value of less than 0.05 was considered statistically significant.

## 5. Conclusions and Limitations of the Study

Conditional deletion of *Sod2* in salivary gland ductal epithelial cells resulted in increased oxidative stress, causing significant changes in gene expression, mitochondrial morphology, and reversible glandular hypofunction. Contrary to our hypothesis, no elevated type I interferon gene signature and development of salivary gland disease matching SjD occurred. Instead, our mouse model’s phenotype matched that noted in non-Sjögren’s sicca patients.

Our study suggests that oxidative stress in ductal cells alone might not be sufficient for SjD development and that additional triggers must be essential for the disease process. However, one of the limitations of our study was the reliance on only the mitochondrial SOD2 pathway for the induction of oxidative stress. Adding *Sod1* deletion to our mouse model would exacerbate cytoplasmic oxidative stress, possibly leading to a severe disease phenotype matching SjD. Another limitation of our study is the lack of data on mitochondrial function in ductal cells with *Sod2* deletion. We expect to undertake these challenging experiments in the future so that molecular mechanisms involved in glandular dysfunction can be discovered.

## Data Availability

The raw data supporting the conclusions of this article will be made available by the authors upon request.

## Supplementary Materials

The following supporting information can be downloaded at: https://www.mdpi.com/article/10.3390/ijms25115983/s1.
