# Supplementary material for: Deleting Mitochondrial Superoxide Dismutase 2 in Salivary Gland Ductal Epithelial Cells Recapitulates Non-Sjögren’s Sicca Syndrome
Source: Int J Mol Sci. 2024 May 30;25(11):5983. doi: 10.3390/ijms25115983 (PMC11172772; doi:10.3390/ijms25115983)
Supplement: Supplementary file 1 [file ijms-25-05983-s001.zip › ijms-2980199-supplementary.pdf]

Supplementary Material:

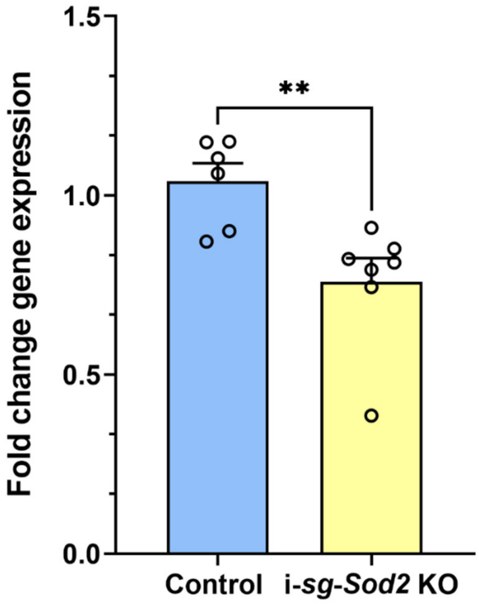

Figure S1. ***Sod2*** **expression is downregulated in the salivary glands of i-sg-*Sod2* KO mice.** Submandibular salivary glands were collected at 12-13 weeks post Tamoxifen treatment. *Hprt1* was used as a housekeeping gene. A pooled salivary gland cDNA sample served as the calibrator, and gene expression was calculated using the 2-ddCT method. Data are represented as fold change over the calibrator. **p<0.01.

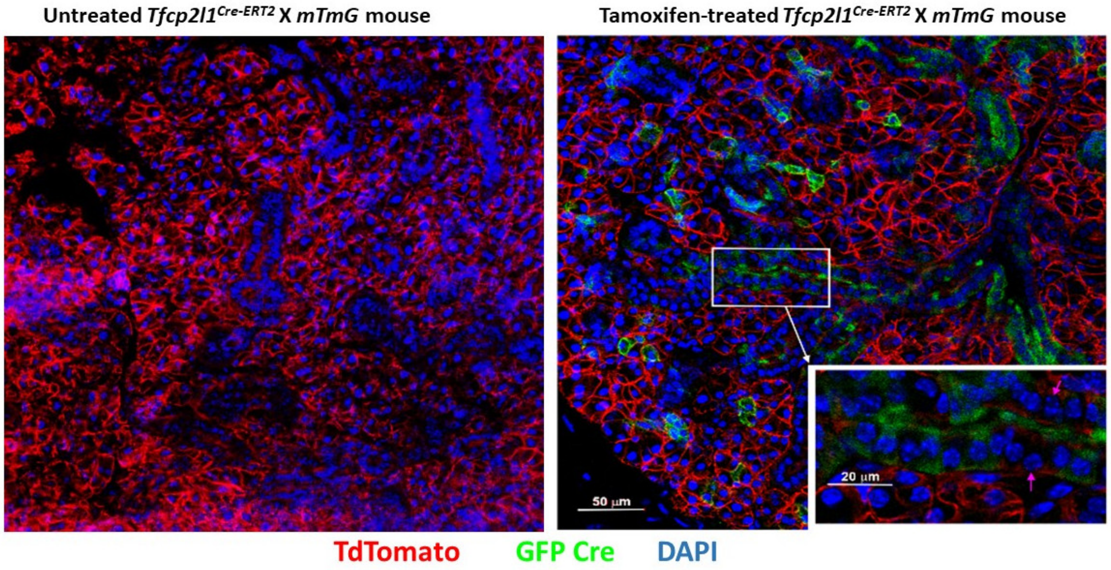

Figure S2. ***Tfcp2l1*** **driven** ***cre/ERT2*** **expression occurs in salivary gland ductal cells.** *Tfcp2l1*$^{cre/ERT2+}$ mice were crossed with mTmG reporter mice to generate *Tfcp2l1*$^{cre/ERT2+}$ x mTmG mice. The F1 mice were either untreated (left panel) or injected with tamoxifen and submandibular salivary glands harvested after 4 weeks. PLP fixed tissue was embedded in OCT, and 10µM sections were evaluated for immunofluorescence. Nuclei were stained with DAPI (blue). Scale bar = 50µM. Inset in the right panel shows an area of tissue section with EGFP+ ductal epithelial cells (scale bar = 20µM)

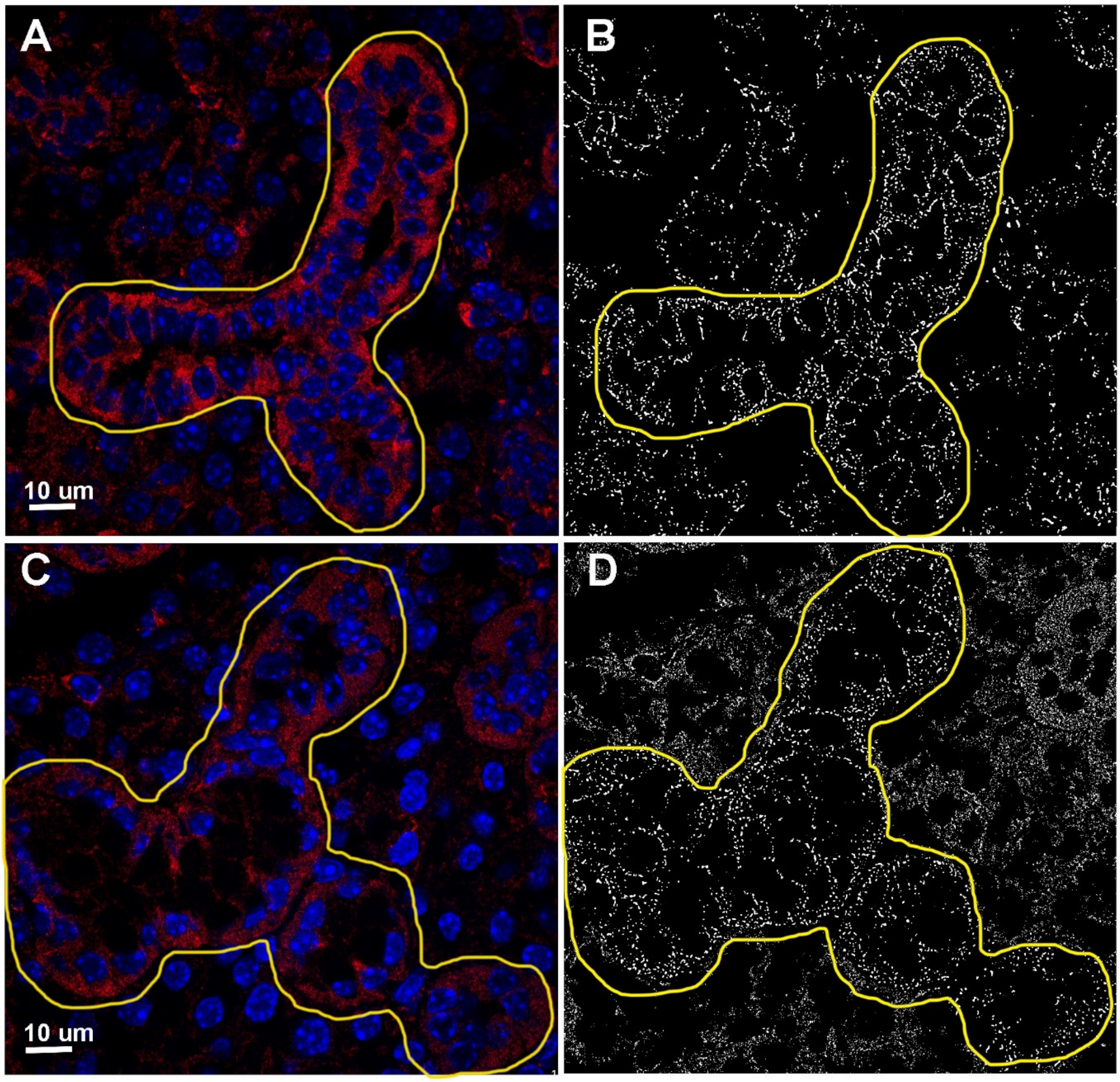

Figure S3. Analysis of mitochondrial structure in salivary gland ductal cells from i-sg-*Sod2* KO mice (A and B) and control littermates (C and D). Salivary gland tissue sections were stained with anti-MFF antibody. Representative images showing MFF staining (**A** and **C**) and thresholded images (**B** and **D**). The ROI is displayed in the area enclosed within the yellow lines. Scale bar, 10μm.
